# Optimality criteria for futility stopping boundaries for group sequential designs with a continuous endpoint

**DOI:** 10.1186/s12874-020-01141-5

**Published:** 2020-11-05

**Authors:** Xieran Li, Carolin Herrmann, Geraldine Rauch

**Affiliations:** 1 Charité – Universitätsmedizin Berlin, corporate member of Freie Universität Berlin, Humboldt-Universität zu Berlin, and Berlin Institute of Health, Institute of Biometry and Clinical Epidemiology, Charitéplatz 1, Berlin, 10117 Germany; 2 Berlin Institute of Health (BIH), Anna-Louisa-Karsch-Str. 2, Berlin, 10178 Germany

**Keywords:** Futility stop, Group sequential design, Continuous endpoint

## Abstract

**Background:**

In clinical trials with fixed study designs, statistical inference is only made when the trial is completed. In contrast, group sequential designs allow an early stopping of the trial at interim, either for efficacy when the treatment effect is significant or for futility when the treatment effect seems too small to justify a continuation of the trial. Efficacy stopping boundaries based on alpha spending functions have been widely discussed in the statistical literature, and there is also solid work on the choice of adequate futility stopping boundaries. Still, futility boundaries are often chosen with little or completely without theoretical justification, in particular in investigator initiated trails. Some authors contributed to fill this gap. In here, we rely on an idea of Schüler et al. (2017) who discuss optimality criteria for futility boundaries for the special case of trials with (multiple) time-to-event endpoints. Their concept can be adopted to define “optimal” futility boundaries (with respect to given performance indicators) for continuous endpoints.

**Methods:**

We extend Schülers’ definition for “optimal” futility boundaries to the most common study situation of a single continuous primary endpoint compared between two groups. First, we introduce the analytic algorithm to derive these futility boundaries. Second, the new concept is applied to a real clinical trial example. Finally, the performance of a study design with an “optimal” futility boundary is compared to designs with arbitrarily chosen futility boundaries.

**Results:**

The presented concept of deriving futility boundaries allows to control the probability of wrongly stopping for futility, that means stopping for futility even if the treatment effect is promizing. At the same time, the loss in power is also controlled by this approach. Moreover, “optimal” futility boundaries improve the probability of correctly stopping for futility under the null hypothesis of no difference between two groups.

**Conclusions:**

The choice of futility boundaries should be thoroughly investigated at the planning stage. The sometimes met, arbitrary choice of futility boundaries can lead to a substantial negative impact on performance. Applying futility boundaries with predefined optimization criteria increases efficiency of group sequential designs. Other optimization criteria than proposed in here might be incorporated.

**Supplementary Information:**

The online version contains supplementary material available at (doi:10.1186/s12874-020-01141-5).

## Background

Conducting clinical trials which fulfil both economical as well as ethical aspects requires extensive efforts in planning. This can be challenging in fixed design clinical trials, as there is no option to react to misspecified planning assumptions during the ongoing trial. Group sequential designs allow for an early stop for either efficacy or futility, thereby, allowing to reduce costs and ethical issues when interim results are either sufficiently convincing or do not justify a further investigation. Group sequential designs are characterized by one or several unblinded interim analyses, thus implying a multiple test problem. Popular methods for alpha adjustment were proposed by Pocock [1] and O’Brien and Fleming [2]. Later, more flexible methods were developed with the idea to define alpha spending functions [3–5]. Following these developments, in the past decades, an increasing number of trials adopted such flexible designs.

Whereas the option for an early efficacy stop is a key feature of group sequential designs, futility stops are not routinely implemented. Stopping a trial early for efficacy implies a successful trial with reduced costs. The probability to stop for efficacy although there is no treatment benefit is naturally controlled by the significance level. In comparison, stopping a trial early for futility means to give up hope for a successful trial based on an interim effect which might have low precision due to small sample sizes at interim. Thereby, the futility stopping boundary is usually defined as a boundary for the interim *p*-value. Valid stopping for futility bounds could reduce costs and avoid involving more patients under unnecessary risks, whereas wrong stopping for futility corresponds to a waste of resources.

Among futility stopping methods of group sequential designs, two main rules are discussed in the literature. Futility stopping rules can either be binding or non-binding, where binding means that stopping is mandatory if the criterion is met and non-binding means that the investigator can freely decide if he or she really wants to stop. Type I error control is guaranteed for both types but there is a decrease in the actual power. In clinical practice, non-binding rules are much more common, as usually it is not only the interim data that affects a decision but also new external data or safety information. When concentrating on binding rules, it is possible to choose larger local significance levels in order to fully exhaust the global significance level [6]. However, this option is usually not applied in practice and more attention should be given to the non-binding option.

There exist sound and broad theoretical methodologies on group sequential designs. In particular, theoretically justified choices of futility stopping boundaries were discussed already decades ago [7, 8]. Several authors [9–12] addressed this issue more generally by defining beta-spending functions in analogy to the well-known alpha-spending functions where the latter take account of the multiplicity issue in group sequential designs. The beta-spending function allows to monitor and control the stage-wise and the global power loss induced by the futility stop.

As additional performance measures for futility boundaries, He et al. [13] referred to the conditional and the predictive power. Gallo et al. [14] more generally discussed performance indicators for choosing futility boundaries including the global power loss, the conditional power, the predictive power, and the probability of correctly stopping for futility under the null hypothesis. In another work of Xi et al. [15], an optimal tuple of the futility boundary and the time point for the interim analysis is determined. This tuple is chosen as a solution of an optimization problem given by an objective function with constraints, where a bound for the power loss defines the constraint and the average sample size defines the performance function. Optimization functions with constraints in the context of adaptive designs have also been recently discussed by Pilz et al. [16]. Instead of formulating constraints, Ondra et al. [17] discuss several adaptive designs by means of optimizing prespecified utility functions. Schüler et al. [18] defined “optimal” futility stopping boundaries under predefined optimality criteria, however for the very special case of (multiple) time-to-event endpoints. Thereby, they rely on the performance measures given by power loss, probability of wrongly stopping for futility and probability of correctly stopping for futility. Whereas approaches based on optimization problems with constraints or maximizing utility functions can be seen as more elegant mathematical solutions, the approach by Schüler et al. [18] might have advantages in the communication to clinical researchers as their basic idea for “optimal” futility boundaries is simply understood: For a given sample size and effect under the alternative, the futility bound which preserves a predefined level of a wrong futility classification is determined. This value serves as a starting value for the “optimal” futility boundary. It is enlarged until the power loss is decreased to an acceptable limit. This defines the “optimal” futility boundary.

Despite these important works, the above reported performance indicators are often not investigated when setting the futility boundary in clinical applications. In particular in investigator initiated trials, futility boundaries are often chosen rather arbitrarily. A common choice in a superiority test setting is a futility boundary of 0.5, where the study is stopped whenever the one-sided interim *p*-value lays above this boundary. This corresponds to the situation of the treatment effect pointing in the wrong direction. For example, the software ADDPLAN which implements sample size recalculation for group sequential designs, sets a default value of 0.5 when a futility stop is included [19]. Moreover, within the R-Package rpact short examples with this futility boundary of 0.5 are provided for illustration [20]. However, this choice of the futility boundary is usually not justified by design performance characteristics. Note however that other sample size calculation software such as nQuery or Pass implement beta-spending functions as a default, so there is no unique standard [21, 22].

In this work, we aim to adopt the approach by Schüler et al. [18] for the more common case of a controlled trial comparing two groups with a continuous endpoint. Whereas for multiple correlated time-to-event endpoints, the findings of optimal futility boundaries can only be realized by simulations, this more simple case allows a straight forward analytical derivation. Using this common and simple design, we aim to contribute to a more profound discussion on futility boundaries in practice and aim to provide an easy understandable and easily applicable tool to overcome the potential gap between developed theory and clinical practice.

This work is structured as follows: In the Methods Section, we introduce the underlying test problem and the group sequential design. Subsequently, we introduce the definition of “optimal” futility boundaries by Schüler et al. [18] adapted to the situation of a continuous primary endpoint. In the Results Section, we first illustrate the concept of the investigated optimality conditions for futility boundaries for the setting of an exemplary clinical trial. Secondly, we compare the performance characteristics of a study with optimally chosen futility boundaries to those with non-optimal boundaries for various design scenarios, where the expression “optimal” in the following refers to the investigated performance criteria. Finally we discuss our results and provide conclusions and implications for future clinical trials.

## Method

Throughout this work, we consider a randomized controlled trial with a continuous primary endpoint which is compared between a new intervention (I) and a control treatment (C)

$$X^{I}_{i} \sim \mathcal{N}\left(\mu^{I},\,\sigma^{2}\right),\ X^{C}_{i} \sim \mathcal{N}\left(\mu^{C},\,\sigma^{2}\right),\ i = 1 \ldots n. $$

For the sake of simplicity, we consider equal standard deviations *σ* and group sizes *n*. The test hypotheses are given in terms of a superiority test

1 $$\begin{array}{@{}rcl@{}} H_{0}:\mu^{I}-\mu^{C}\leq0 \ \text{versus} \ H_{1}:\mu^{I}-\mu^{C}>0. \end{array} $$

Thereby, without loss of generality, a larger value of the endpoint is assumed to be favorable.

### Group sequential design

We consider a group sequential design with two sequences, that is with one interim analysis. The total maximal sample size is *N*=2·*n*, the total interim sample size is denoted by *N*_1_=2·*n*_1_. The interim test statistic can be formulated as

2 $$\begin{array}{@{}rcl@{}} T_{1}:=\frac{\bar{X}_{1}^{I}-\bar{X}_{1}^{C}}{S_{pooled,1}}\cdot\sqrt{\frac{n_{1}}{2}}, \end{array} $$

with observed interim means $\bar {X}_{1}^{I}, \bar {X}_{1}^{C}$ and a pooled standard deviation at interim *S*_*pooled*,1_. This test statistic corresponds to the normal approximation test for continuous endpoints.

The study is stopped for efficacy at the interim stage in case the one-sided interim *p*-value *p*_1_ is smaller than or equal to the adjusted local one-sided significance level *p*_1_≤*α*_1_.

The study is stopped for futility if *p*_1_>*α*_0_, where *α*_0_ is the futility boundary.

If the trial is not stopped within the interim analysis, then additional *N*_2_=*N*−*N*_1_ patients are recruited. The test statistic for the final analysis is then given by

3 $$\begin{array}{@{}rcl@{}} T_{1+2}:=\frac{w_{1}\cdot T_{1}+w_{2}\cdot T_{2}}{\sqrt{w_{1}^{2}+w_{2}^{2}}}, \end{array} $$

where *T*_2_ is the independent incremental test statistic including exclusively the data of the second stage and *w*_1_,*w*_2_ are predefined weights which must be fixed at the planning stage. This is also known as the inverse normal combination test [23] as the stage-wise test statistics can be written as the inverse of the normal distribution function applied to the stage-wise *p*-values *T*_*i*_=*Φ*^−^(*p*_*i*_), *i*=1,2. The combination of *p*-values provided by the inverse normal method is just one option among others to combine the stage-wise *p*-values. Another famous approach would be the use of the Fisher combination test [24]. The idea presented in here is also transferable when using another combination function.

A common way to choose the above weights in the inverse normal combination function is to define ${w_{1}=\sqrt {n_{1}}}$ and $w_{2}=\sqrt {n_{2}}$.

The null hypothesis *H*_0_ is rejected at the final analysis if the corresponding *p*-value is smaller than or equal to the adjusted local one-sided significance level *p*_1+2_≤*α*_1+2_. The key idea of the inverse normal approach is that by constructing the final test statistic *T*_1+2_ from the independent stage-wise test statistics *T*_1_ and *T*_2_, the covariance of the joint distribution of *T*_1_ and *T*_1+2_ is

$$\begin{array}{@{}rcl@{}} Cov\left(T_{1},T_{1+2}\right)=\sqrt{\frac{n_{1}}{n}}, \end{array} $$

and thus the joint distribution is fully specified.

The local significance levels for the interim analysis and the final analysis can be specified such that the overall type I error is controlled, that is

4 $$\begin{array}{@{}rcl@{}} P_{H_{0}}\left(p_{1} \leq \alpha_{1} \cup \left(\alpha_{1} < p_{1} \cap p_{1+2} \leq \alpha_{1+2}\right)\right) = \alpha. \end{array} $$

If binding futility stopping boundaries are applied, the futility boundary *α*_0_ can be incorporated in the above equation to obtain larger optimized local significance levels *α*_1_ and *α*_1+2_. We will not consider this option, as even if a fixed futility stopping rule is incorporated in the trial protocol, there are often external reasons to make exceptions from this binding rule, which is not a problem as long as the local significance levels are chosen according to Eq. (4).

The local significance levels *α*_1_ and *α*_1+2_ can be derived using various existing methods, such as constant levels as proposed by Pocock [1], increasing levels as given by O’Brien-Flemming [2], or flexible alpha spending functions as e.g. described by Lan and DeMets [4]. In our work, for the sake of simplicity, we rely on Pocock boundaries that is *α*_1_=*α*_1+2_. The remaining question is how to choose an adequate value of *α*_0_ already at the planning stage.

### Optimality criteria for futility boundaries

The idea of “optimal” futility boundaries proposed by Schüler et al. [18] is to assure a high probability to stop correctly for futility. This means stopping when there is only no or a non-relevant treatment effect, while simultaneously controlling the loss in power and the probability of correctly stopping for futility when in fact, the underlying treatment effect is relevant. In the following, we will use the term “optimal” with respect to these criteria. As discussed in the introduction, there are however various other performance indicators and different approaches to quantify the total performance. Therefore, optimality is not a unique perspective and we do not intend to present the “best” solution. In the following, assume that the trial is powered to detect a standardized effect ${\Delta =\frac {\mu ^{I}-\mu ^{C}}{\sigma }}$ with power 1−*β* at a global one-sided significance level of *α*. To introduce the concept of optimal futility boundaries, some additional parameters are required: Let *Pow*_loss_<1−*β* denote the admissible overall power loss caused by applying a binding futility boundary. Moreover, the probability to wrongly stop for futility when in fact the underlying standardized treatment effect is given by the relevant effect *Δ* should be limited by *π*_wrong_∈[0,1]. Using these notations, a futility boundary fulfils the so called **admissible conditions** [18] if the following requirements are satisfied:

1. *P*_*Δ*_(*p*_1_>*α*_0_)≤*π*_wrong_,
2. *P*_*Δ*_(*p*_1_≤*α*_1_∪(*α*_1_<*p*_1_<*α*_0_∩*p*_1+2_≤*α*_1+2_))≥1−*β*−*Pow*_loss_.

Note that the concept of the optimality parameter *Pow*_loss_ is similar to the beta-spending approach proposed by several authors [9–12]. The beta-spending approach allows to control the stage-wise power loss induced by futility stopping boundaries. In contrast, we exclusively focus on the global power loss. Note that both approaches guarantee a limited (stage-wise) power loss only for the assumed effect *Δ*. For smaller effects the power loss can become unacceptably high. Therefore, we strongly recommend to choose *Δ* as the *minimal* clinically relevant effect and not as the expected effect.

In the following, any futility boundary fulfilling the admissible conditions will be denoted as *α*_0,ad_. Note that for a clinical trial with a continuous endpoint and the design specifications given above, the first admissible condition can be translated into

$$\alpha_{0,\text{ad}}\geq 1-\Phi\left(z_{\pi_{\text{wrong}}}+\Delta\cdot\sqrt{\frac{n_{1}}{2}}\right), $$

where *Φ*(∗) denotes the distribution function of the standard normal distribution and *z*_(∗)_ denotes the corresponding quantile of the standard normal distribution. The second admissible condition is equivalent to

$$\begin{array}{@{}rcl@{}} &&1-\Phi\left(z_{1-\alpha_{1}}-\Delta\cdot\sqrt{\frac{n_{1}}{2}}\right)\\ &&+MV_{\mathbf{\mu,\Sigma}}\left(z_{1-\alpha_{1}}, z_{1-\alpha_{1+2}}\right)\\ &&-MV_{\mathbf{\mu,\Sigma}}\left(z_{1-\alpha_{0,\text{ad}}}, z_{1-\alpha_{1+2}}\right)\\ &\geq&1-\beta-Pow_{\text{loss}}, \end{array} $$

where *MV*_**μ,Σ**_(∗) is the distribution function of the multivariate normal distribution with expectation

$$\begin{array}{@{}rcl@{}} \pmb{\mu}=\left(\Delta\cdot \sqrt{\frac{n_{1}}{2}};\Delta\cdot \sqrt{\frac{n}{2}}\right) \end{array} $$

and variance-covariance matrix

$$\begin{array}{@{}rcl@{}} \mathbf{\Sigma}= \left(\begin{array}{ll} \sqrt{\frac{n_{1}}{n}} & 1 \\ 1 & \sqrt{\frac{n_{1}}{n}} \\ \end{array}\right). \end{array} $$

For predefined parameters *Pow*_loss_ and *π*_wrong_, there exists a whole set of admissible futility boundaries fulfilling the above conditions. Only the probability of correctly stopping for futility is left to further optimize an admissible futility stopping boundary. As the probability to correctly stop for futility increases with decreasing futility boundary, this implies that the optimal futility boundary *α*_0,opt_ is the minimum over the set of all admissible futility boundaries *α*_0,ad_. With this definition, we can compute the optimal futility boundary at the planning stage of a clinical trial analytically. However, it can happen that the actual achievable probability to correctly stop for futility is still considered as too small. In this case, it might be reasonable to choose slightly larger values of *Pow*_loss_ and *π*_wrong_.

## Results

Given predefined design parameters, the optimal futility boundaries can be analytically computed at the planning stage. An R-function which calculates the “optimal” futility boundary for arbitrary design parameters is provided as online supplementary material (see Additional File 1).

### A clinical trial example

In the following, we will illustrate the benefit of using an optimal futility boundary compared to an arbitrary choice of a futility boundary by means of a real clinical trial example.

The ChroPac-Trial [25] is a blinded, randomized, controlled clinical trial. The primary endpoint is the quality of life of patients with chronic pancreatitis 24 months after surgical interventions. The intervention group receives a duodenum-preserving pancreatic head resection and is compared to a control group receiving pancreatoduodenectomy. The aim is to show superiority of the intervention. The primary endpoint is measured by the quality of life questionnaire EORTC QLQ-C30, which provides a score for physical functioning. The score ranges from 0 to 100 with a higher score indicating a better quality of life. Although a score is generally seen as an ordinal endpoint, it is a common approach to treat a score with a large range as a continuous endpoint. A score difference of 10 is considered as a clinically relevant treatment difference and 20 is assumed to be the common standard deviation.

The trial was planned to detect a standardized treatment effect $\Delta =\frac {10}{20}=0.5$ at a one-sided global significance level *α*=0.025 with power 1−*β*=0.90. This results in a total sample size of 172 patients (86 per group) when the null hypothesis is tested with a standard t-test for independent groups. Note that the original trial was planned with a fixed design. For illustrative purposes, we will now apply a group sequential design to illustrate the new concept.

Applying a two-stage group sequential design with an interim look after 50% of the patients being fully observed and local adjusted significance levels according to Pocock with *α*_1_=*α*_1+2_=0.0147, the above sample size yields a power of 0.88. In order to apply the concept of an optimal futility boundary now, we need specifications of *Pow*_loss_ and *π*_wrong_. A power loss caused by futility stopping of *Pow*_loss_=0.05 is considered reasonable. The probability to wrongly stop for futility should of course be small. Thus, we may choose *π*_wrong_=0.05. With these parameter settings, the optimal futility boundary is given by *α*_0,opt_=0.22.

It is also common to anticipate a power of 0.80. Therefore, as a reference design, we will also calculate the optimal futility boundary for the above setting when the global maximal sample size of the group sequential design is only *N*=140, which results in a power of 0.80 without stopping for futility. In this case, the optimal futility boundary is given by *α*_0,opt_=0.33.

**Fig. 1:**
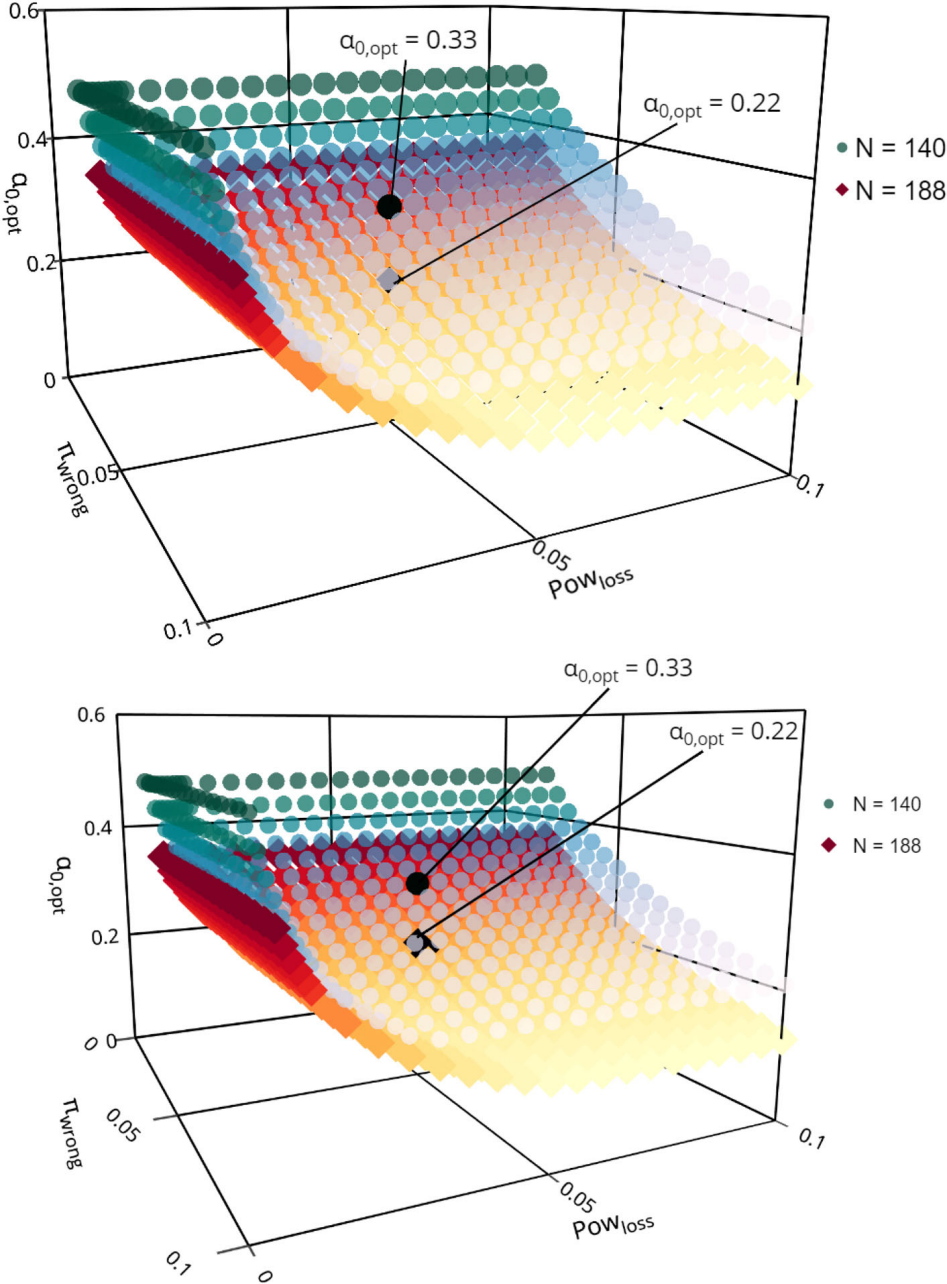
The “optimal” futility boundary *α*_0,opt_ as a function of the admissible parameters *Pow*_*loss*_ and *π*_*wrong*_ for *N*=140 (blue dots) and *n*=188 (red squares). The black symbols highlight the “optimal” futility boundaries for *Pow*_loss_=0.05 and *π*_wrong_=0.05

The two admissible parameters, power loss *Pow*_*loss*_ and the probability of wrongly stopping for futility *π*_*wrong*_, determine jointly the optimal futility boundary *α*_0,opt_. Therefore, *α*_0,opt_ can be displayed as a function of these two parameters as illustrated in Fig. 1, which allows to investigate graphically how the optimal futility boundary changes when the admissible parameters are varied.

From Fig. 1 it can nicely be seen that the optimal futility boundary also depends on the sample size, where a larger sample size results in a smaller futility boundary. It can be seen that for *N*=140, the optimal futility boundary is mainly determined by the parameter *π*_wrong_, whereas for *N*=188 the influence of *Pow*_loss_ grows. In order to quantitatively assess the impact of variations of the admissible parameter settings, Table 1 shows the resulting optimal futility boundaries for selected parameter values of *Pow*_loss_ and *π*_wrong_ for both sample size settings *N*=188 and 140.

**Table 1:** Performance characteristics for the group sequential design with “optimal” futility boundaries based on different admissible condition parameters for *N*=182 and *N*=140. The last lines in the two sample size settings show the performance characteristics for the arbitrary choice of *α*_0_=0.5

Sample	Admissible condition		“Optimal” futility	Actual	Probability of wrongly	Probability of correctly	
size	parameters		boundary	power	stopping for futility	stopping for futility	
*n*	*Pow*_loss_	*π*_wrong_	*α*_0,opt_	under *Δ*=0.5	$P_{\Delta _{\text {true}}=0.5}\left (p_{1}>\alpha _{0}\right)$	$P_{\Delta _{\text {true}}=0.25}\left (p_{1}>\alpha _{0}\right)$	$P_{\Delta _{\text {true}}=0.0}\left (p_{1}>\alpha _{0}\right)$
188	0.01	0.01	0.46	0.90	0.01	0.13	0.54
	0.05	0.01	0.46	0.90	0.01	0.13	0.54
	0.01	0.05	0.29	0.89	0.03	0.26	0.71
	0.05	0.05	0.22	0.89	0.05	0.33	0.78
	0.01	0.10	0.29	0.89	0.03	0.26	0.71
	0.05	0.10	0.13	0.85	0.10	0.47	0.87
	0.0013	0.008	0.50	0.90	0.01	0.11	0.50
140	0.01	0.01	0.59	0.80	0.01	0.10	0.41
	0.05	0.01	0.59	0.80	0.01	0.10	0.41
	0.01	0.05	0.33	0.79	0.05	0.27	0.67
	0.05	0.05	0.33	0.79	0.05	0.27	0.67
	0.01	0.10	0.32	0.79	0.05	0.28	0.68
	0.05	0.10	0.21	0.77	0.10	0.41	0.79
	0.0013	0.018	0.50	0.80	0.02	0.15	0.50

Column 1 displays the underlying sample size. Columns 2 and 3 show the specification of the admissible condition parameters *Pow*_*loss*_ and *π*_*wrong*_. The resulting optimal futility boundary is displayed in Column 4. Columns 5 to 8 show the performance of the design by various performance measures such as the actually achieved power including stopping for futility (Column 5), the probability of wrongly stopping for futility under the anticipated relevant effect *Δ* (Column 6), as well as the probability of correctly stopping for futility under a small non-relevant effect, which is half the size of the anticipated effect and under the null hypothesis with no effect (Columns 7 and 8).

It can be seen from Table 1 that a very low value of *π*_wrong_=0.01 results in high optimal futility boundaries, which may be close to the often arbitrarily chosen value of *α*_0_=0.5 (Row 1) or even larger (Row 8). However, the probability of correctly stopping for futility is relatively low in these scenarios.

Looking at the parameter settings where the resulting probability of correctly stopping for futility under either the null hypothesis or half of the relevant treatment effect is at least above 20%, it can be deduced that a slightly larger value of, e.g. *π*_wrong_=0.05, is a better choice.

The admissible power loss *Pow*_loss_ is generally often not exhausted, especially for smaller values of *π*_wrong_. For example, a change in the parameter *Pow*_loss_ does not have an impact on the optimal futility boundary when *π*_wrong_ is fixed to either 0.01 or 0.05 for *N*=140.

Note that a conventional choice of the futility boundary is *α*_0_=0.5. Looking at Table 1 it can be seen that for the favorable settings, where the probability of correctly stopping for futility is not too small and lays above 20%, the optimal futility boundaries are considerably smaller than the conventional choice of *α*_0_=0.5. For *N*=188 the optimal futility boundaries range between *α*_0,opt_=0.13 and *α*_0,opt_=0.46, for *N*=140 between *α*_0,opt_=0.21 and *α*_0,opt_=0.59.

## Discussion

Although efficacy boundaries in group sequential designs are widely discussed in the literature, the choice of futility boundaries gains much less attention in clinical applications. A naive choice choice of a futility boundary of *α*_0_=0.5, where an interim *p*-value of *p*_1_>0.5 suggests an early stopping for futility, means that at interim, as soon as the treatment effect points into the adverse direction, the trial is stopped. Although this is intuitive, the implications of this futility boundary choice on the design performance are not always investigated. However, the choice of the futility boundary naturally influences the power of the study design. Moreover, a large futility boundary implies that the probability to stop the study, when indeed there is no or only a non-relevant effect (correct stopping for futility), can be small. In contrast, a too low futility boundary can imply that the probability of wrongly stopping for futility, when there is a relevant treatment effect, is considered as too large.

Some authors have proposed adaptive design strategies to optimize design parameters, like the value of the futility boundary and the number and timing of interim looks [11, 13, 14, 16, 17]. Thereby, different concept were proposed, e.g. optimization problems with constraints [14, 16] or maximization of utility functions [17]. A comparison between these different approaches is still lacking. The optimality criteria initially proposed by Schüler et al. [18] allow to define a relatively simple concept of “optimal” futility boundaries, which was originally proposed in the context of (composite) time-to-event endpoints, by balancing the performance characteristics of global power loss and the probability of correctly and wrongly stopping for futility. The task of this work was to adapt this concept to the more general case of a group sequential design with a continuous endpoint. We showed that with the concept of optimal futility boundaries, it is possible to quantify the performance characteristics and the implications of a futility boundary already at the planning stage. By a clinical trial example, we demonstrated that arbitrarily choosing *α*_0_=0.5 can lead to very unfavorable performance characteristics in some situations. However, there are also trial settings, where the choice of *α*_0_=0.5 is close to or even smaller than the optimal one. This highlights the necessity to investigate the implications of different futility stopping boundaries already at the planning stage.

The concept of optimal futility boundaries fits the regulatory guidance documents provided by the U.S. Food and Drug Administration [26] and European Medicines Agency [27] for confirmatory trials. If a trial sponsor aims at applying our method in a confirmatory trial, the power loss and probability of wrongly and correctly stopping for futility can be predefined as two additional design parameters in the clinical trial protocol. Simulations are not required as the operating characteristics can be derived analytically for continuous endpoints. An R-code providing the analytical solution is provided as online supplementary material (see Additional File 1). Thus, the design modifications are easily calculated and communicated which is a requirement of the FDA guidance [26].

A possible limitation of the presented futility concept is that the choice of the admissible condition parameters, which limits the power loss and controls the probability of wrongly stopping for efficacy, is to a certain extend arbitrary. We therefore recommend to calculate the optimal futility boundaries for a range of plausible admissible condition parameters and to investigate the performance characteristics. In particular, the probability of correctly stopping for futility should be reasonably high (above 20% as a rule of thumb). This approach can lead to a reasonable choice of the futility boundary that provides a fair balance between the different performance characteristics.

In this work, we concentrated on a two-stage group sequential design with a continuous endpoint with local significance levels adjusted according to Pocock [1]. The corresponding R-source code (see Additional File 1) can be easily adapted to use other alpha spending functions and other *p*-value combination tests. Moreover, the concept can equivalently be adopted to binary endpoints, which will be the task of future work.

An attractive argument for the presented approach lays in the simplicity of the key idea. In particular within investigator initiated trials, there often exist not theoretically founded recommendations for choosing futility bounds. One main aim of this article is thus to encourage the theoretical justification of futility boundaries in practical applications. There are different ways to do so of which our approach is only one option.

## Conclusions

While other trial design parameters and operational characteristics are routinely investigated in the planning stage of group sequential designs, futility boundaries should not be neglected. The concept of an “optimal” futility boundary method as introduced in here allows to control the power loss and the probability of wrongly stopping for futility, while maximizing the probability of correctly stopping for futility. We recommend to investigate futility boundaries following our approach over a range of parameter settings and to carefully compare the resulting futility boundaries to the arbitrary choice of *α*_0_=0.5 when planning a trial with a group sequential design.

## Supplementary Information

**Additional file 1** The file contains the R-source code for the calculation of the “optimal” futility boundary for a predefined design setting and admissible parameters.

## Data Availability

The R-source code for the calculation of the optimal futility boundary is available (see Additional File 1).
