# Correction to: Optimality criteria for futility stopping boundaries for group sequential designs with a continuous endpoint

**DOI:** 10.1186/s12874-020-01161-1

**Published:** 2020-11-25

**Authors:** Xieran Li, Carolin Herrmann, Geraldine Rauch

**Affiliations:** 1 Charité – Universitätsmedizin Berlin, corporate member of Freie Universität Berlin, Humboldt-Universität zu Berlin, and Berlin Institute of Health, Institute of Biometry and Clinical Epidemiology, Charitéplatz 1, 10117 Berlin, Germany; 2 Berlin Institute of Health (BIH), Anna-Louisa-Karsch-Str. 2, 10178 Berlin, Germany

**Correction to: BMC Med Res Methodol 20, 274 (2020)**

**https://doi.org/10.1186/s12874-020-01141-5**

Following publication of the article [1], it was noted that due to a typesetting error the old version of Fig. 1 is displayed with the correct version of Fig. 1, and it should be deleted.

The correct Fig. 1 and caption have been included in this correction, and the original article has been corrected.

**Fig. 1:**
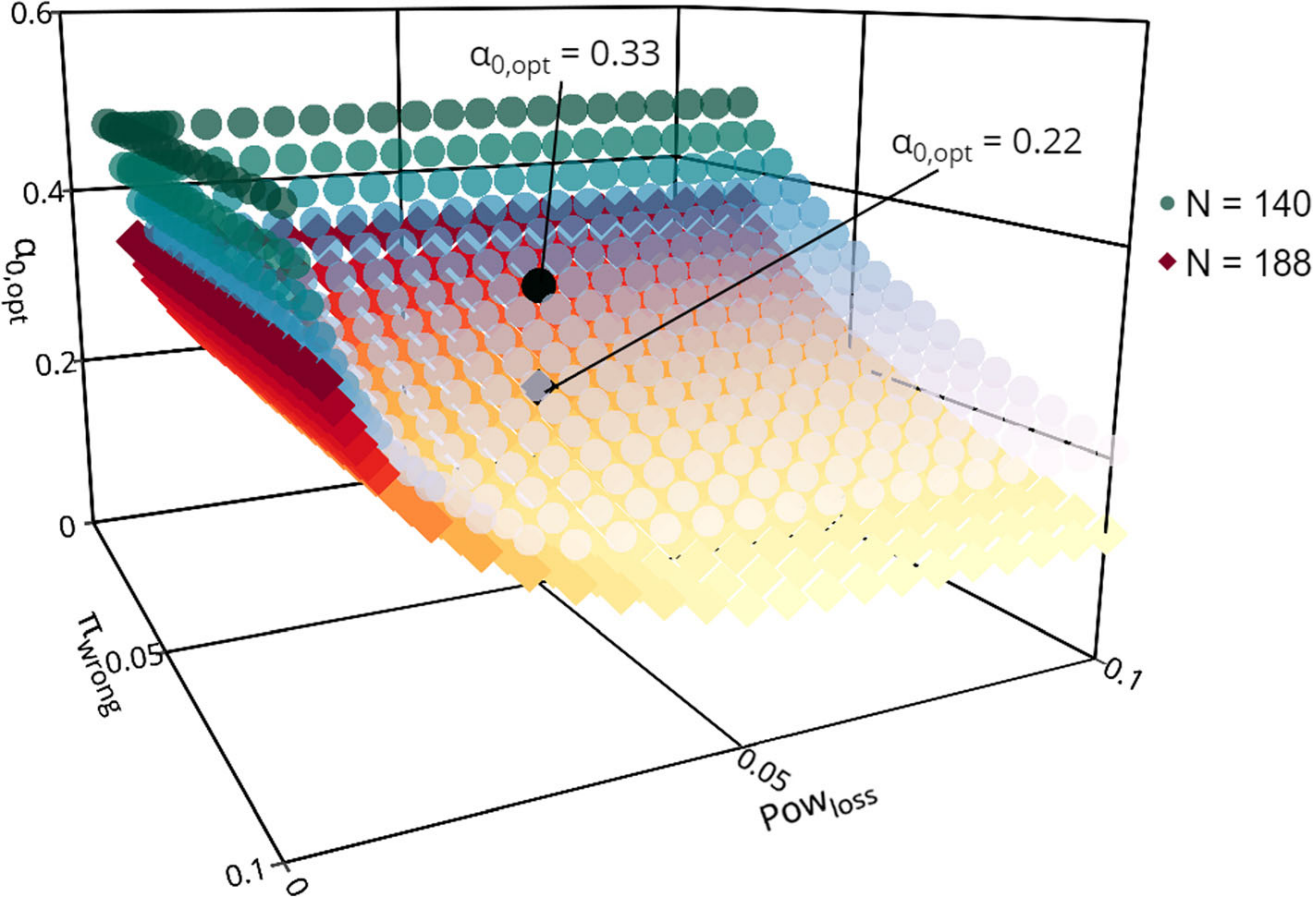
The “optimal” futility boundary *α*_0,opt_ as a function of the admissible parameters *Pow*_*loss*_ and *π*_*wrong*_ for *N* = 140 (blue dots) and *n* = 188 (red squares). The black symbols highlight the “optimal” futility boundaries for *Pow*_loss_ = 0.05 and *π*_wrong_ = 0.05
