# Supplementary material for: Optimality criteria for futility stopping boundaries for group sequential designs with a continuous endpoint
Source: BMC Med Res Methodol. 2020 Nov 5;20:274. doi: 10.1186/s12874-020-01141-5 (PMC7643306; doi:10.1186/s12874-020-01141-5)
Supplement: Supplementary file 1 — Additional file 1 The file contains the R-source code for the calculation of the “optimal” futility boundary for a predefined design setting and admissible parameters. [file 12874_2020_1141_MOESM1_ESM.pdf]

```
#######################################################################################
#####
#             optimal futility bound program v1.0                                 #
# Package dependency:                                                             #
#   mvtnorm                                                               #
#                                                                       #
# Input:                                                                  #
#   delta:          effect size                                           #
#   n12:            total sample size                                       #
#   alpha_1:         efficacy boundary at stage 1                            #
#   alpha_2:         efficacy boundary at stage 2/final                        #
#   infofrac:        information fraction                                 #
#   power_loss:      power loss (admissible condition parameter)                  #
#   pi_wrong:        probability of wrongly stopping (admissible condition parameter)#
#   precision:       precision for the futility boundary. default 1E-5             #
#                                                                       #
# Output:                                                                 #
#   power_loss:      power loss (as input)                                 #
#   pi_wrong:        probability of wrongly stopping (as input)                  #
#   alpha0_opt:      optimal futility boundary                              #
#   prob_wrong_fut:   probability of wrongly stopping for futility                  #
#   prob_halfdel_fut: probability of correctly stopping for futility under half delta #
#   prob_zerodel_fut: probability of correctly stopping for futility under 0 delta    #
#                                                                       #
#######################################################################################
#load mvtnorm package
library(mvtnorm)


#define the core function optimal_fut_bound
optimal_fut_bound <- function(delta, n_12, alpha_1, alpha_2, infofrac, power_loss, pi_wrong,
precision = 1E-5){
```

```
  #test statistic at stage 1 and stage 2/final
  T_1 <- delta*sqrt((n_12*infofrac)/4)
  T_12 <- delta*sqrt(n_12/4)

  #covariance of the joint distribution T_1 and T_12
  cov_mtx <- matrix(c(1,sqrt(infofrac), sqrt(infofrac), 1), ncol=2)

  #beta = 1- power of two-stage group sequential design with efficacy boundaries alpha_1 and
alpha_2
  beta <- pmvnorm(lower = c(-Inf,-Inf),
                  upper = c(qnorm(1-alpha_1),qnorm(1-alpha_2)),
                  mean  = c(T_1,T_12),
                  sigma = cov_mtx)
  #bisectional search for alpha0, the futility boundary starting bounded between
  #wrongly stopping at stage 1 (lower) and no stopping (upper), with initial precision of 1
  alpha0_upper <- 1
  alpha0_lower <- 1 - pnorm(qnorm(pi_wrong) + T_1)
  prec <- 1

  while(prec > precision){
    #search start with the middle point at each step
    alpha0_opt <- (alpha0_lower + alpha0_upper) / 2

    #actual power for search
    power_actual <- 1 - pnorm(qnorm(1-alpha_1), T_1, 1) +
                     pmvnorm(lower = c(qnorm(1-alpha0_opt), qnorm(1-alpha_2)),
                             upper = c(qnorm(1-alpha_1),Inf),
                             mean  = c(T_1,T_12),
                             sigma = cov_mtx)
    #update the search area to be bounded by the searched point either as the upper or the lower
limit
    ifelse(power_actual < 1 - beta - power_loss, alpha0_lower <- alpha0_opt, alpha0_upper <-
alpha0_opt)
```

```
    #update the searched precision
    prec <- alpha0_upper - alpha0_lower
  }
  #further performance characteristics
  prob_wrong_fut   <- pnorm(qnorm(1-alpha0_opt) - T_1)
  prob_halfdel_fut <- pnorm(qnorm(1-alpha0_opt) - 0.5*T_1)
  prob_zerodel_fut <- pnorm(qnorm(1-alpha0_opt))

  return(list("power_loss"       = power_loss,
              "pi_wrong"         = pi_wrong,
              "alpha0_opt"       = alpha0_opt,
              "power_actual"     = power_actual,
              "prob_wrong_fut"   = prob_wrong_fut,
              "prob_halfdel_fut" = prob_halfdel_fut,
              "prob_zerodel_fut" = prob_zerodel_fut)
        )
}
#The following code can be used to create Table 1
for (pi_wrong in c(0.01,0.05,0.1)){
  for (power_loss in seq(0.01,0.05,0.04)){
    x <- optimal_fut_bound(delta = 0.5, n_12 = 188, alpha_1 = 0.0147, alpha_2 = 0.0147, infofrac = 0.5,
power_loss, pi_wrong)
    print(paste("Powloss = ", power_loss, ", piwrong = ", format(pi_wrong, nsmall=2), ", alpha0_opt= ",
format(round(x$alpha0_opt,2), nsmall=2),
                ", ActualPower = ", format(round(x$power_actual, 2), nsmall=2), ", Pwrong = ",
format(round(x$prob_wrong_fut, 2), nsmall=2),
                ", Pcorrect1/2 = ", format(round(x$prob_halfdel_fut, 2), nsmall=2),", Pcorrect0 = ",
format(round(x$prob_zerodel_fut, 2), nsmall=2)))
  }
}
```
